# Attitudes towards Enhanced Recovery after Surgery (ERAS) interventions in colorectal surgery: nationwide survey of Australia and New Zealand colorectal surgeons

**DOI:** 10.1007/s00423-022-02488-7

**Published:** 2022-03-11

**Authors:** James Wei Tatt Toh, Geoffrey Peter Collins, Nimalan Pathma-Nathan, Toufic El-Khoury, Alexander Engel, Stephen Smith, Arthur Richardson, Grahame Ctercteko

**Affiliations:** 1 Discipline of Surgery, Sydney Medical School, The University of Sydney, Sydney, Australia; 2 Colorectal Department, Division of Surgery and Anaesthetics, Westmead Hospital, Cnr Hawkesbury and Darcy Rd, Westmead, Sydney, NSW 2145 Australia; 3 The University of Notre Dame, Sydney, Australia; 4 Colorectal Department, Royal North Shore Hospital, Sydney, Australia; 5 Colorectal Department, John Hunter Hospital, Newcastle, Australia; 6 Upper Gastrointestinal Department, Westmead Hospital, Sydney, Australia

**Keywords:** Enhanced recovery, ERAS, Colorectal surgery, Surgical site infection

## Abstract

**Background:**

Whilst Enhanced Recovery after Surgery (ERAS) has been widely accepted in the international colorectal surgery community, there remains significant variations in ERAS programme implementations, compliance rates and best practice recommendations in international guidelines.

**Methods:**

A questionnaire was distributed to colorectal surgeons from Australia and New Zealand after ethics approval. It evaluated specialist attitudes towards the effectiveness of specific ERAS interventions in improving short term outcomes after colorectal surgery. The data were analysed using a rating scale and graded response model in item response theory (IRT) on Stata MP, version 15 (StataCorp LP, College Station, TX).

**Results:**

Of 300 colorectal surgeons, 95 (31.7%) participated in the survey. Of eighteen ERAS interventions, this study identified eight strategies as most effective in improving ERAS programmes alongside early oral feeding and mobilisation. These included pre-operative iron infusion for anaemic patients (IRT score = 7.82 [95% *CI*: 6.01–9.16]), minimally invasive surgery (IRT score = 7.77 [95% *CI*: 5.96–9.07]), early in-dwelling catheter removal (IRT score = 7.69 [95% *CI*: 5.83–9.01]), pre-operative smoking cessation (IRT score = 7.68 [95% *CI*: 5.49–9.18]), pre-operative counselling (IRT score = 7.44 [95% *CI*: 5.58–8.88]), avoiding drains in colon surgery (IRT score = 7.37 [95% *CI*: 5.17–8.95]), avoiding nasogastric tubes (IRT score = 7.29 [95% *CI*: 5.32–8.8]) and early drain removal in rectal surgery (IRT score = 5.64 [95% *CI*: 3.49–7.66]).

**Conclusions:**

This survey has demonstrated the current attitudes of colorectal surgeons from Australia and New Zealand regarding ERAS interventions. Eight of the interventions assessed in this study including pre-operative iron infusion for anaemic patients, minimally invasive surgery, early in-dwelling catheter removal, pre-operative smoking cessation, pre-operative counselling, avoidance of drains in colon surgery, avoiding nasogastric tubes and early drain removal in rectal surgery should be considered an important part of colorectal ERAS programmes.

**Supplementary Information:**

The online version contains supplementary material available at 10.1007/s00423-022-02488-7.

## Introduction

Enhanced Recovery after Surgery (ERAS) programmes have been shown to improve morbidity, recovery and hospital length of stay (LOS) in both laparoscopic and open colorectal surgery [1, 2]. Whilst there are established American and European ERAS guidelines [3, 4], there are significant variations in guidelines internationally, and there have been no local guidelines to guide surgeons performing colorectal surgery in Australia and New Zealand. Whilst Australian guidelines do recommend implementation of ERAS programmes in colorectal cancer surgery [5], best practice parameters have yet to be established in Australia and New Zealand.

As part of any ERAS programme, ERAS compliance has been shown to be associated with fewer complications and shorter LOS [6]. However, the merit of specific elements of ERAS protocols is more difficult to measure [6, 7]. Some elements of ERAS have more robust evidence in the surgical literature supporting their recommendation. For other interventions, there is limited data or despite abundant level 1 evidence, there remains a dichotomy of views, such as for mechanical bowel preparation (MBP) and oral antibiotics (OAB). In any case, significant variation exists in the implementation of ERAS.

The aim of this study was to evaluate the current attitudes and perspectives amongst specialist colorectal surgeons in Australia and New Zealand regarding ERAS interventions. We provide a Likert Scale analysis with item response theory (IRT) statistical modelling to rank the ERAS interventions in order of importance, and provide a recommendation based on specialist colorectal surgeons’ opinions and attitudes for the interventions that should be considered an important part of any ERAS programme.

## Materials and methods

A questionnaire on ERAS was distributed to colorectal surgeons in Australia and New Zealand who are current members of the Colorectal Surgical Society of Australia and New Zealand (CSSANZ). The survey received institutional board approval (2019/ETH11810). ERAS Society guidelines, American Society of Colorectal Surgeons (ASCRS) guidelines and American College of Surgeons National Surgical Quality Improvement Programme (NSQIP) data variables were used to inform the elements of ERAS assessed in this survey [3, 4, 8], of which eighteen ERAS interventions were evaluated in this survey. These included preoperative counselling, smoking cessation; preoperative iron or blood transfusion to correct anaemia; MBP; OAB alone; MBP and OAB; preoperative carbohydrate loading; preoperative immunonutrition; postoperative laxative use; avoidance of nasogastric tube (NGT); use of epidural for open surgery; use of epidural for minimally invasive surgery; minimally invasive surgery; early removal of drains for rectal surgery; avoidance of drains in colon surgery; early removal of urinary catheter within 1 to 2 days for rectal surgery, within 1 day for colon surgery; use of selective non-steroidal anti-inflammatory drugs (NSAIDs) as part of multimodal pain management and use of non-selective NSAIDs as part of multimodal pain management..

Other interventions such as preoperative prophylaxis against thrombosis, control of intra-operative body temperature, prehabilitation, perioperative nausea and vomiting prophylaxis, intraoperative fluid management, sham feeding, cessation of alcohol, medical optimisation of chronic disease, preoperative prophylaxis against infection, preoperative prophylaxis against thrombosis and control of intraoperative body temperature have been described in parts in the surgical literature but were not examined in this survey. Early oral feeding and mobilisation are key concepts of fast track surgery since it was first described by Kehlet in 1997 [9] and were not questioned in this survey as these were considered standard of care in the delivery of all fast track ERAS programmes.

The surgeons were asked to evaluate the components of ERAS in terms of how likely they were to improve short-term (30-day) outcomes including LOS and readmission rates (refer to Appendix 1). Surgeons who did not initially respond to the survey were prompted on two more occasions before the survey was closed. Some results from this questionnaire were first published in 2021 [10] but the study mainly focused on practice patterns and attitudes towards MBP and OAB in colorectal surgery. In this study, however, results of the survey were adapted to focus on the attitudes and perspectives towards ERAS interventions in colorectal surgery and to compare these attitudes with the evidence in the surgical literature.

The questionnaire used a 10-scale Likert score for each question, which was used to assess the attitudes of surgeons towards the effectiveness of well-established ERAS strategies. A Likert score of eight to 10 was considered definitely or very likely to be effective. Six to seven was considered effective or somewhat effective, five was considered neutral, three to four was considered not really effective and zero to two was considered very likely not effective or definitely not effective.

Each question was analysed and ranked using a rating scale and graded response model in item response theory (IRT) on Stata/MP, version 15 (StataCorp LP, College Station, TX). IRT is a helpful tool commonly used in the scoring of questionnaires and surveys; it scales individual responses according to the respondent’s overall level of performance compared with other respondents.

## Results

**Fig. 1:**
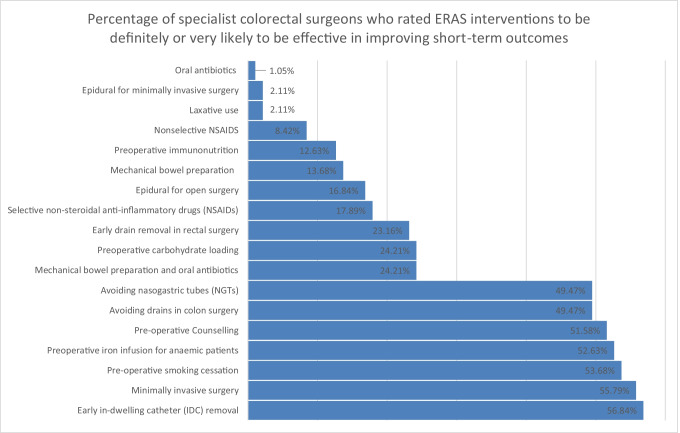
Percentage of specialist colorectal surgeons who rated specific ERAS interventions to be definitely or very likely to be effective in improving short-term outcomes

Of 300 colorectal surgeons in Australia and New Zealand, 95 (31.7%) responded to the survey. Summary statistics and weighted averages were calculated for each ERAS intervention where relevant (Table 1). The percentage of surgeons responding definitely or very likely to be effective (Likert score 8–10) for each intervention is shown in Fig. 1.

**Table 1:** Specialist attitudes towards Enhanced Recovery after Surgery (ERAS) interventions and specialist attitude towards effectiveness in improving short-term outcomes

Enhanced Recovery after Surgery (ERAS) interventions	Weighted mean	% Likert score 8–10	IRT score	Lower limit (95% *CI*)	Upper limit (95% *CI*)
Preoperative iron infusion for anaemic patients	7.84	53%	7.82	6.01	9.16
Minimally invasive surgery	7.78	56%	7.77	5.96	9.07
Early in-dwelling catheter (IDC) removal	7.65	57%	7.69	5.83	9.01
Pre-operative smoking cessation	7.51	54%	7.68	5.49	9.18
Pre-operative counselling	7.47	52%	7.44	5.58	8.88
Avoiding drains in colon surgery	7.24	49%	7.37	5.17	8.95
Avoiding nasogastric tubes (NGTs)	7.21	49%	7.29	5.32	8.8
Preoperative carbohydrate loading	6.11	24%	IRT modelling not possible	-	-
Early drain removal in rectal surgery	5.71	23%	5.64	3.49	7.66
Mechanical bowel preparation and oral antibiotics	5.34	24%	5.39	2.99	7.48
Preoperative immunonutrition	4.96	13%	4.85	2.92	6.48
Selective non-steroidal anti-inflammatory drugs (NSAIDs)	4.79	18%	4.68	2.49	6.76
Mechanical bowel preparation	4.78	14%	4.87	2.8	6.78
Epidural for open surgery	4.59	17%	4.67	2.47	6.76
Nonselective NSAIDS	3.26	8%	IRT modelling not possible	-	-
Laxative use	3.17	2%	IRT modelling not possible	-	-
Oral antibiotics	3.06	1%	IRT modelling not possible	-	-
Epidural for minimally invasive surgery	1.76	2%	IRT modelling not possible	-	-

### ERAS components ranked by weighted mean and IRT score

IRT modelling was used to statistically analyse the questionnaire results and was able to be applied in 13 of the 18 ERAS interventions examined. Eight interventions achieved an IRT score > 5.5. Of the eighteen ERAS interventions, pre-operative iron infusion was ranked first (weighted mean = 7.84, IRT = 7.82 [95% *CI*: 6.01–9.16]). This was followed by minimally invasive surgery (weighted mean = 7.78, IRT score = 7.77 [95% *CI*: 5.96–9.07]), early in-dwelling catheter removal (weighted mean = 7.65, IRT score = 7.69 [95% *CI*: 5.83–9.01]), pre-operative smoking cessation (weighted mean = 7.51, IRT score = 7.68 [95% *CI*: 5.49–9.18]), pre-operative counselling (weighted mean = 7.47, IRT score = 7.44 [95% *CI*: 5.58–8.88]), avoiding drains in colon surgery (weighted mean = 7.24, IRT score = 7.37 [95% *CI*: 5.17–8.95]), avoiding NGTs (weighted mean = 7.21, IRT score = 7.29 [95% *CI*: 5.32–8.8]) and early drain removal in rectal surgery (weighted mean = 5.71, IRT score = 5.64 [95% *CI*: 3.49–7.66]). An IRT score was unable to be modelled for preoperative carbohydrate loading (weighted mean = 6.11) due to significant discontinuous regions relating to a significant dichotomy of opinion amongst specialists.

Of the remaining strategies, MBP and OAB (weighted mean = 5.34, IRT score = 5.39 [95% *CI*: 2.99–7.48]) were considered a more effective strategy when compared to MBP alone (weighted mean = 4.78, IRT score = 4.87 [95% *CI*: 2.8–6.78]) and OAB alone with no bowel preparation (weighted mean 3.06, IRT modelling not possible). OAB alone, despite promising evidence in the surgical literature [11, 12], was not considered an intervention associated with better outcomes. Preoperative immunonutrition (weighted mean = 4.96, IRT score = 4.85 [95% *CI*: 2.92–6.48]), selective NSAIDs (weighted mean = 4.79, IRT score = 4.68 [95% *CI*: 2.49–6.76]) and epidural for open surgery (weighted mean = 4.59, IRT score = 4.67 [95% *CI*: 2.47–6.76]) were considered neutral.

In addition to OAB alone, three interventions were considered not likely to be effective or definitely not effective: laxative use (weighted mean = 3.17, IRT modelling not possible), epidural for minimally invasive surgery (weighted mean = 1.76, IRT modelling not possible) and non-selective NSAIDs (weighted mean = 3.26, IRT modelling not possible). IRT modelling was not possible for these lower ranked ERAS interventions due to discontinuous modelling.

### ERAS components ranked by percentage % Likert score 8–10 (definitely or very likely to be effective).

Seven interventions were reported by half or more of colorectal surgeons to be definitely or very likely to be effective in improving short term outcomes (% Likert score 8–10) (Fig. 1). These included early IDC removal (56.84%), minimally invasive surgery (55.79%), pre-operative smoking cessation (53.68%), pre-operative iron infusion for anaemic patients (52.63%), pre-operative counselling (51.58%), avoiding drains in colon surgery (49.47%) and avoiding NGTs (49.47%).

MBP and OAB (% Likert score 8–10 = 24.21%) were considered a better strategy than MBP (% Likert score 8–10 = 13.68%) and OAB alone without bowel preparation (% Likert score 8–10 = 3.06%).

## Discussion

It is important to develop best practice parameters for ERAS in colorectal surgery in Australia and New Zealand. To date, this is the largest survey of specialist attitudes towards ERAS in Australia and New Zealand.

In this study, eight interventions of the 18 examined were considered by specialist colorectal surgeons to improve short-term outcomes in colorectal surgery (IRT score > 5.5). These include pre-operative iron infusion for anaemic patients, minimally invasive surgery, early indwelling catheter (IDC) removal, pre-operative smoking cessation, pre-operative counselling, avoidance of drains in colon surgery, avoiding NGTs and early drain removal in rectal surgery. In order to understand the results of this survey, it is important to evaluate the attitudes of the colorectal surgeons in the context of the existing evidence within the surgical literature.

### Preoperative iron infusion for anaemic patients

Within the surgical literature, in patients who are iron deficient, preoperative intravenous iron infusion has been shown to reduce hospital LOS and the need for blood transfusion [9, 13]. ERAS Society guidelines recommend pre-operative iron infusions in iron deficient patients and avoidance of peri-operative blood transfusions [4]. In patients with anaemia of chronic disease who are not iron deficient, intravenous (but not oral) iron is still efficacious in the management of preoperative anaemia [14]. In this survey, preoperative iron infusion for anaemic patients was ranked as one of the most important ERAS interventions in improving short-term outcomes.

### Minimally invasive surgery

Minimally invasive surgery is one of the mainstay elements of ERAS. It has become a key component of ERAS in both colon and rectal surgery [15–19], with these studies reporting improved recovery, LOS, blood loss and complication rates. The oncological outcome associated with minimally invasive surgery is comparable to open surgery with the long term follow-up of the CLASICC trial demonstrating no significant differences in overall survival and local recurrence between open and minimally invasive colon and rectal surgery [20]. However, two more recent randomised trials (ALaCaRT and ACOSOG Z6051) using pathologic specimen quality as primary outcome measures following rectal surgery failed to demonstrate non-inferiority of laparoscopic surgery [21, 22] and future studies on oncological outcomes achieved with minimally invasive surgery may influence the recommendations on minimally invasive surgery in rectal cancer management.

### Early in-dwelling catheter removal

It is widely believed that early removal of urinary catheters result in lower rates of urinary tract infection (UTI) at the expense of higher rates of acute urinary retention (AUR), with catheter duration of > 2 days found to be associated with twice the risk of UTI [23]. However, AUR can usually be managed successfully with in–out catheterisation. An observational ERAS study demonstrated low (14%) rates of AUR in patients undergoing colorectal surgery under an established ERAS protocol [24]. Another observational ERAS study linked early removal of urinary catheter with reduced LOS on multivariate analysis [25]. Surgeons in this survey supported the guideline recommendations for urinary catheter removal at 48 h following pelvic surgery, compared with 24 h following colonic surgery [3].

### Smoking cessation

Smoking cessation interventions include behavioural modification and pharmacotherapy. These interventions vary widely by mode of delivery, duration and intensity [26]. There has not been an abundance of evidence for smoking cessation interventions in colorectal surgery. A 2003 randomised controlled trial with 60 patients failed to find a significant benefit for counselling and nicotine replacement therapy [27]. Two systematic reviews from 2011 in a broader surgical population found that interventions initiated more than 4 to 6 weeks prior to surgery reduced the rates of wound and pulmonary complications [28, 29]. However, a subsequent Cochrane review and meta-analysis in 2014 found that intensive interventions initiated at least 4 weeks prior to surgery reduced the rate of wound but not pulmonary complications [26]. Brief behavioural and pharmacotherapy interventions have been associated with only a modest impact on smoking cessation prior to surgery but had no statistically significant impact on perioperative complications [26]. No studies in the literature have reported any serious adverse effects relating to perioperative smoking intervention. Whilst the literature provides only a weak recommendation for smoking cessation, smoking cessation at least 2 weeks prior to surgery was seen as one of the most important interventions by colorectal surgeons who participated in this survey.

### Preoperative counselling

Preoperative counselling may be in the form of person-to-person counselling, audiovisual resources and smartphone-based applications [30]. Outcomes measured in the literature have included LOS, readmission, morbidity, pain, mobility, anxiety, patient distress, patient satisfaction and quality of life. In a scoping review from 2020, positive results were reported for most counselling interventions relating to LOS. Person-to-person counselling was found to be most effective in reducing LOS, with less benefit associated with smart phone, tablet and audiovisual interventions [30]. In addition, an RCT comparing an enhanced recovery programme versus standard care in colorectal patients found that accurate perioperative information and ongoing guidance made an independent contribution to LOS [31]. Preoperative counselling was considered an important ERAS intervention in this survey.

### Avoidance of nasogastric tubes

In the surgical literature, routine NGT decompression is not recommended in colorectal surgery [3]. Studies thus far have not shown any difference in nausea, vomiting, wound infection or intestinal obstruction with routine use of NGTs [32, 33]. Additionally, NGTs delay the time to oral intake by 2 days and carry an increased risk of pharyngolaryngitis [34–36].

### Avoidance of drains in colon surgery

Guidelines have recommended against the routine use of peritoneal drains in colon surgery [3, 4] based on a substantial body of literature evaluating peritoneal drains after colon surgery [37–40]. A 2016 meta-analysis of 11 RCTs on pelvic and peritoneal drains found no increase in the rate of anastomotic leakage, mortality, wound infection or reoperation rates associated with drains [38]. The use of drains may rarely be associated with post-operative bowel obstruction, colocutaneous fistula, enterocutaneous fistula and skin ulceration [41–43]. Colorectal surgeons in this survey agree with guideline recommendations to avoid drains in colon surgery.

### Early removal of drains in rectal surgery

The evidence behind the use of drains in rectal surgery is equivocal with several guidelines recommending against the routine use of pelvic drains in rectal surgery [3, 4]. A 2016 meta-analysis on pelvic and peritoneal drains found no difference in the rate of anastomotic leakage, mortality, wound infection or reoperation rates with and without drains [38]. A subsequent RCT demonstrated that the use of a pelvic drain after rectal surgery conferred no benefit even for anastomoses below the peritoneal reflection [37]. However, a 2019 systematic review and meta-analysis on the use of pelvic drains following anterior resection concluded that whilst drains did not improve overall complication rate or outcomes following anastomotic leakage, there was a three-fold reduction in mortality observed in the group of patients with pelvic drains [44]. The study findings were that drains did not reduce leaks or complication rates but reduced mortality. Whilst the literature does not provide strong recommendations on drains following rectal surgery, colorectal surgeons considered early removal of drains in rectal surgery an important ERAS intervention in this survey.

### Mechanical bowel preparation and oral antibiotics

The debate on MBP and OAB continues. The World Health Organisation (WHO), ASCRS and the Society of American Gastrointestinal and Endoscopic Surgeons (SAGES) support the use of MBP and OAB [3, 45]. However, the ERAS Society and Australian guidelines on MBP and OAB still do not recommend its use [4, 5]. In this survey, specialist colorectal surgeons in Australia and New Zealand considered MBP and OAB as the most effective of the bowel preparation strategies [10]. This may be because there is now abundant level 1 and 2 evidence for the use of MBP and OAB to reduce the rate of SSIs. A network meta-analysis from 2018 comparing MBP and OAB, OAB alone, MBP alone and no MBP found MBP and OAB to have the greatest effect on reduction in SSI (with OAB alone coming in second), but found no difference in the rates of anastomotic leak, readmission or reoperation between any groups [12]. A systematic review and meta-analysis from 2019 compared MBP and OAB with MBP alone and found a significant reduction in SSI, anastomotic leak, 30-day mortality, overall morbidity and ileus in the MBP and OAB group [11]. When MBP and OAB was compared to OAB alone, there was no significant difference in SSI or rates of anastomotic leak; however, there was a significant reduction in 30-day mortality and post-operative ileus in the MBP and OAB group. There was insufficient evidence in the literature to compare MBP and OAB to no preparation, OAB alone to no preparation and OAB to MBP. A subsequent RCT from 2020 (ORALEV) compared OAB alone to no preparation and found a significant reduction in SSI in the OAB group [46].

Despite some evidence for its use, OAB alone was ranked very poorly in this study and was not considered an intervention that many surgeons would adopt. This may be because there are few studies reporting on OAB alone [12] and, despite promising benefits shown in recent studies [46], more scientific evidence may be required before this would be considered by surgeons as an acceptable approach. Furthermore, many surgeons believe that bowel preparation in rectal surgery improves bowel handling [47], especially during difficult pelvic dissection and stapling.

### Carbohydrate loading

Carbohydrate loading is recommended by several guidelines in non-diabetic patients [3, 4]. Carbohydrate drinks have been shown to improve insulin resistance, post-operative gastrointestinal function and overall well-being [48, 49]. However, the effect on LOS and post-operative complications is less certain. Although a 2014 systematic review and meta-analysis found that carbohydrate loading reduced LOS compared to placebo or fasting; this effect disappeared when comparing to placebo only (not fasting) [50], similar to the findings of a network meta-analysis in 2017 [51]. The specialists in this survey were divided on the effectiveness of carbohydrate loading on improving short-term outcomes.

### Immunonutrition

Although immunonutrition does not feature consistently in major ERAS guidelines, perioperative immunonutrition is recommended by the ERAS Society guidelines [3, 4]. Altered nitric oxide synthase and T-cell dysfunction in the context of tissue injury following major surgery have been found to cause acute arginine depletion [52]. Although formulations vary, most immunonutrition contains arginine, nucleotides and omega-3 fatty acids. A 2018 systematic review and meta-analysis of RCTs on the use of immunonutrition in colorectal cancer patients undergoing surgery demonstrated a significant reduction in infectious complications (primarily SSI) and improved LOS [53]. ESPEN guidelines have recommended perioperative immunonutrition for malnourished cancer patients undergoing major surgery [54]. Despite reasonable evidence in the literature, specialist colorectal surgeons in this survey did not have a strong view on immunonutrition and further research is required.

### Non-steroidal anti-inflammatory drugs

Several guidelines have identified non-steroidal anti-inflammatory drugs (NSAIDs) as the key opioid-sparing component of multimodal analgesia [3, 4] and there is evidence that their use reduces the time to flatus and stool [55]. However, their use remains controversial due to a possible association with anastomotic leakage [56–59]. Non-selective NSAIDs are likely to be higher risk than selective NSAIDs [58]. The intravenous, non-selective agent ketolorac has been associated with increased risk of anastomotic leak [60]. There may also be a higher risk of anastomotic leak associated with NSAIDs in patients undergoing emergency colorectal surgery [61]. Specialists in this survey did not consider non-selective NSAID an effective ERAS intervention. There was more acceptance of selective NSAIDs than non-selective NSAIDs but attitudes towards NSAIDs were divided in this survey.

### Thoracic epidural anaesthesia

Thoracic epidural anaesthesia has been part of guideline recommendations for open colorectal surgery but not for routine use in laparoscopic surgery [3, 4]. Epidural has traditionally been the gold standard for analgesia following open abdominal surgery compared with patient-controlled analgesia (PCA) or systemic opioids [62, 63]. Compared with continuous wound infusion, epidural has superior pain control at the expense of increase rates of post-operative hypotension [64]. However, multiple RCTs have shown that epidural may increase LOS in minimally invasive colorectal surgery [65–67], likely due to the increased incidence of post operative hypotension and UTI [68].

This survey demonstrated that epidural in laparoscopic colorectal surgery was not considered to be an effective intervention in improving short-term outcomes. Furthermore, there was not strong support for epidural in this survey in open colorectal surgery despite strong evidence in the literature for its use. This may be related to the risk of post-operative complications and the emergence of alternative modalities such as continuous wound infusions of local anaesthetic.

### Laxatives

Laxatives have recently been considered as part of a multimodal strategy to reduce the risk of post-operative ileus [4]. A 2020 systematic review of RCTs found that routine post-operative laxative use after major abdominal surgery reduced time to passage of stool but found no difference in LOS [69]. A subsequent RCT compared laxatives vs. no laxatives in colorectal patients and again reported a decrease in post-operative ileus but demonstrated no difference in LOS or post-operative complications [70]. In this survey, laxative use was considered ineffective in improving short-term outcomes. Further research is required on the use of laxatives in reducing the risk of ileus.

### Limitations

The survey was only able to achieve a response rate of 31.7% (95 of 300 Australian and New Zealand colorectal surgeons). However, this is comparable to the response rate of the European survey of colorectal surgeons (40.2% (426/1059)) [71], and better than the survey of US colorectal surgeons which achieved a response rate of 11.2% (359/3206) [72]. This survey had a significantly better response rate than a previous Australian survey on bowel preparation prior to colorectal surgery [47].

## Conclusion

This survey has demonstrated the current perspectives and attitudes of colorectal surgeons from Australia and New Zealand regarding ERAS interventions. Alongside core fast track concepts of early oral feeding and mobilisation, eight of the interventions assessed in this study including pre-operative iron infusion for anaemic patients, minimally invasive surgery, early IDC removal, pre-operative smoking cessation, pre-operative counselling, avoidance of drains in colon surgery, avoiding NGTs and early drain removal in rectal surgery should be considered an important part of colorectal ERAS programmes.

## Supplementary Information

Below is the link to the electronic supplementary material.

Supplementary file1 (DOCX 257 KB)

## Data Availability

The datasets generated during and/or analysed during the current study are available from the corresponding author on reasonable request.
