# Supplementary material for: Attitudes towards Enhanced Recovery after Surgery (ERAS) interventions in colorectal surgery: nationwide survey of Australia and New Zealand colorectal surgeons
Source: Langenbecks Arch Surg. 2022 Mar 11;407(4):1637–46. doi: 10.1007/s00423-022-02488-7 (PMC9283181; doi:10.1007/s00423-022-02488-7)
Supplement: Supplementary file 1 — Supplementary file1 (DOCX 257 KB) [file 423_2022_2488_MOESM1_ESM.docx]

Effectiveness of ERAS interventions

**In your opinion, how effective are these interventions in providing better short term (30-day) outcomes and / or improved length of stay / readmission rates following colorectal surgery?**

1. Preoperative counselling


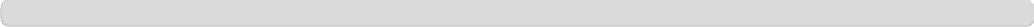

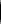

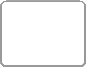

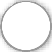


0 (not at all)

5 (neutral)

10 (definitely)

1. Smoking cessation at least 2 weeks prior to surgery


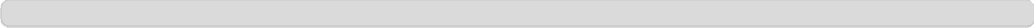

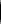

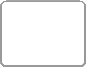

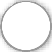


0 (not at all)

5 (neutral)

10 (definitely)

1. Preoperative iron or blood transfusion to correct anaemia


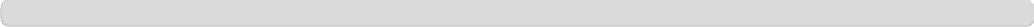

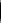

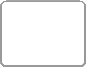

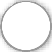


0 (not at all)

5 (neutral)

10 (definitely)

1. Mechanical bowel preparation alone (no oral antibiotics) vs. no preparation


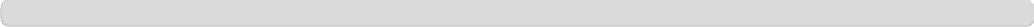

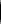

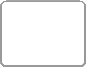

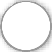


0 (not at all)

5 (neutral)

10 (definitely)

1. Oral antibiotics alone (no bowel preparation) vs. no preparation


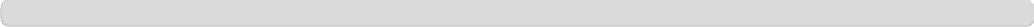

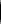

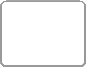

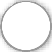


0 (not at all)

5 (neutral)

10 (definitely)

1. Mechanical bowel preparation and oral antibiotics vs. no preparation


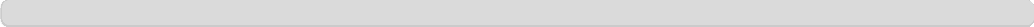

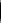

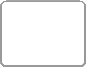

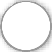


0 (not at all)

5 (neutral)

10 (definitely)

1. Preoperative carbohydrate loading


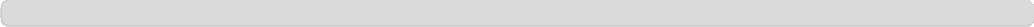

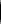

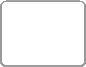

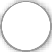


0 (not at all)

5 (neutral)

10 (definitely)

1. Preoperative immunonutrition


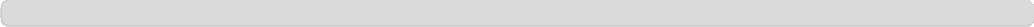

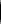

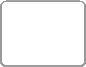

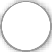


0 (not at all)

5 (neutral)

10 (definitely)

1. Postoperative laxative use


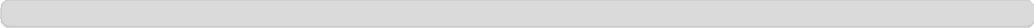

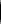

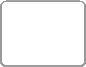

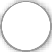


0 (not at all)

5 (neutral)

10 (definitely)

1. Avoidance of nasogastric tube


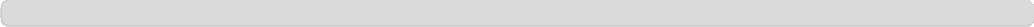

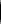

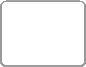

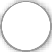


0 (not at all)

5 (neutral)

10 (definitely)

1. Use of epidural for open surgery


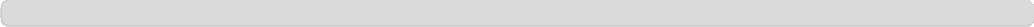

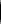

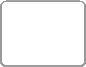

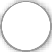


0 (not at all)

5 (neutral)

10 (definitely)

1. Use of epidural for minimally invasive surgery


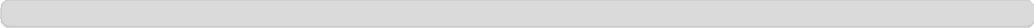

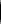

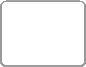

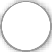


0 (not at all)

5 (neutral)

10 (definitely)

1. Minimally invasive surgery (when compared to open surgery)


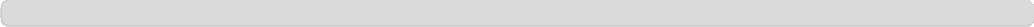

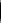

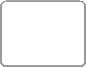

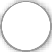


0 (not at all)

5 (neutral)

10 (definitely)

1. Early removal of drain (within 1-2 days) in the post-operative period for rectal surgery


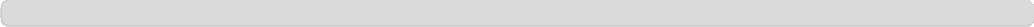

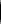

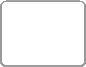

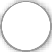


0 (not at all)

5 (neutral)

10 (definitely)

1. Avoidance of drain in colon surgery


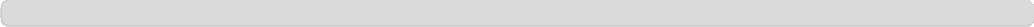

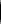

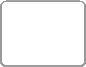

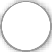


0 (not at all)

5 (neutral)

10 (definitely)

1. Early removal of urinary catheter within one to two days for rectal surgery; within one day for colon surgery


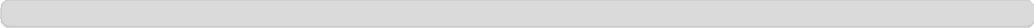

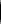

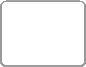

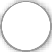


0 (not at all)

5 (neutral)

10 (definitely)

1. Use of selective NSAIDS as part of multimodal pain management


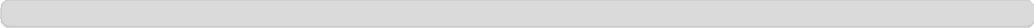

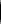

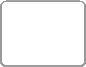

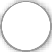


0 (not at all)

5 (neutral)

10 (definitely)

1. Use of non-selective NSAIDs as part of multimodal pain management


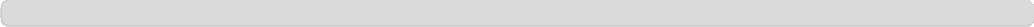

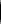

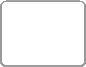

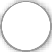


0 (not at all)

5 (neutral)

10 (definitely)
